# DNA damage response in a 2D-culture model by diffusing alpha-emitters radiation therapy (Alpha-DaRT)

**DOI:** 10.1038/s41598-024-62071-6

**Published:** 2024-05-20

**Authors:** Hitomi Nojima, Atsushi Kaida, Yusuke Matsuya, Motohiro Uo, Ryo-ichi Yoshimura, Lior Arazi, Masahiko Miura

**Affiliations:** 1 Department of Dental Radiology and Radiation Oncology, Graduate School of Medical and Dental Sciences, Tokyo Medical and Dental University, 1-5-45 Yushima, Bunkyo-ku, Tokyo, 113-8549 Japan; 2 Nuclear Science and Engineering Center, Japan Atomic Energy Agency, 2-4 Shirakata, Tokai, Ibaraki 319-1195 Japan; 3 Faculty of Health Sciences, Hokkaido University, Kita-12 Nishi-5, Kita-ku, Sapporo, Hokkaido 060-0812 Japan; 4 Department of Advanced Biomaterials, Graduate School of Medical and Dental Sciences, Tokyo Medical and Dental University, 1-5-45 Yushima, Bunkyo-ku, Tokyo, 113-8549 Japan; 5 Department of Radiation Therapeutics and Oncology, Graduate School of Medical and Dental Sciences, Tokyo Medical and Dental University, 1-5-45 Yushima, Bunkyo-ku, Tokyo, 113-8549 Japan; 6 Unit of Nuclear Engineering, Faculty of Engineering Sciences, Ben-Gurion University of the Negev, P.O.B. 653, 8410501 Be’er-Sheva, Israel

**Keywords:** Cancer therapy, Radiotherapy, Cell biology

## Abstract

Diffusing alpha-emitters radiation therapy (Alpha-DaRT) is a unique method, in which interstitial sources carrying ^224^Ra release a chain of short-lived daughter atoms from their surface. Although DNA damage response (DDR) is crucial to inducing cell death after irradiation, how the DDR occurs during Alpha-DaRT treatment has not yet been explored. In this study, we temporo-spatially characterized DDR such as kinetics of DNA double-strand breaks (DSBs) and cell cycle, in two-dimensional (2D) culture conditions qualitatively mimicking Alpha-DaRT treatments, by employing HeLa cells expressing the Fucci cell cycle-visualizing system. The distribution of the alpha-particle pits detected by a plastic nuclear track detector, CR-39, strongly correlated with γH2AX staining, a marker of DSBs, around the ^224^Ra source, but the area of G2 arrested cells was more widely spread 24 h from the start of the exposure. Thereafter, close time-lapse observation revealed varying cell cycle kinetics, depending on the distance from the source. A medium containing daughter nuclides prepared from ^224^Ra sources allowed us to estimate the radiation dose after 24 h of exposure, and determine surviving fractions. The present experimental model revealed for the first time temporo-spatial information of DDR occurring around the source in its early stages.

## Introduction

Alpha particles have several distinct features which make them an attractive option for radiation therapy^1^. Their high linear energy transfer (LET)—approximately 100–200 keV/μm in water—leads to a much higher yield of double strand breaks (DSBs) compared to low-LET irradiation, as well as to clustered DNA damage within several base pairs which are recognized as complex DNA lesions^2,3^. This high-LET further makes alpha particles effective against hypoxic cells which have elevated resistance to low-LET treatments, and their cell-killing efficacy is less sensitive to the cell-cycle stage^1^. Alpha-emitting atoms, bound to targeting vectors (e.g., ligands to prostate-specific membrane antigen^4^), are typically used systematically, with the aim of treating single cancer cells or micro-metastatic disease^5–7^.

**Figure 1:**
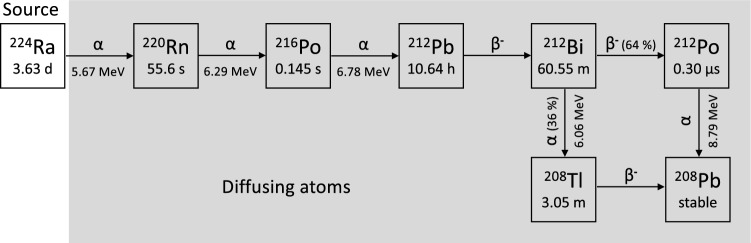
^224^Ra decay chain. Data are taken from NuDat3 database (available from a website at https://www.nndc.bnl.gov/nudat3/).

Diffusing alpha-emitters Radiation Therapy (“Alpha-DaRT”)^8^ aims to utilize alpha particles for the treatment of solid tumors. Alpha-DaRT employs interstitial sources loaded with low activities (a few μCi; 1 μCi = 3.7 × 10^4^ Bq) of ^224^Ra (half-life 3.63 d). To overcome the short range of alpha particles in tissue (< 0.1 mm), the sources are designed to release from their surface a series of short-lived alpha-emitting daughter radionuclides, which diffuse over a few mm around each source, killing cells through their alpha decays. The ^224^Ra decay chain is shown in Fig. 1. The net effect of the diffusion process is the enhancement of the effective therapeutic range of alpha particles from a few dozen μm to a few mm.

Several preclinical studies have been performed to determine appropriate clinical designs for Alpha-DaRT^8–13^. In particular, to provide quantitative tumor dose estimates, an approximate theoretical framework—the Diffusion-Leakage model—was introduced^14^, studied for realistic source configurations^14–17^, and numerically validated^18^. Theoretical modeling combined with measured diffusion patterns in treated tumors led to a choice of 7.4 × 10^4^ Bq ^224^Ra per source and a nominal inter-source spacing of 5 mm as a starting point for clinical trials on squamous cell carcinoma (SCC). Two clinical trials have been completed and reported. The first was conducted in Israel and Italy for primary and recurrent cases of skin and head and neck SCC. Short-term follow-up resulted in a complete response (CR) rate of approximately 80%. However, subsequently, patients with CR showed 60% local progression-free survival after 1 year of observation^19^. A recently reported trial for recurrent or unresectable skin cancer in the United States showed a 100% CR rate 12 weeks after treatment^20^. Adverse effects in both studies were mild to moderate, with no indication of radiation damage to surrounding healthy tissue or distant organs. These clinical results demonstrate that Alpha-DaRT could be a promising modality of radiation therapy. However, given the fact that some cases initially showing CR had subsequent recurrence, improving the dose coverage could further enhance the treatment efficacy. Indeed, clinical designs of 1.1 × 10^5^ Bq/source and a ~ 4 mm source interval were successfully employed in the clinical trial conducted in the United States in 2023^20^.

Early preclinical animal studies using Alpha-DaRT were related to dose determination around the source within the tumor tissues where necrosis was a marker of treatment effect. Recent studies were related to combination with other drugs including chemotherapeutic agents, angiogenesis inhibitors, and immune checkpoint inhibitors^21–23^. Although extensive preclinical and clinical Alpha-DaRT studies have been performed, DNA damage response (DDR)—thought to be crucial to inducing cell death—has never been studied.

DDR including cell cycle checkpoint and DNA repair occurs shortly after DNA damage, influencing subsequent cell death or survival. Therefore, exploring DDR around the source could potentially provide clinically useful insights. The primary purpose of this study was to characterize such DDR in an in vitro monolayer system exposed to an Alpha-DaRT source. The secondary goal was to determine the radiosensitivity of the studied cells using a colony-formation assay.

To study DDR, we employed a combination of three experimental methods: (1) the cell cycle-visualizing system Fucci, which allows differentiating between cells in the G1 phase and cells in the S/G2/M phases, emitting red and green fluorescence, respectively^24^ (a technique with which we have extensive experience from previous X-ray studies^25,26^); (2) γH2AX staining as a marker of DSBs; and (3) CR-39—a plastic nuclear track detector which allows quantifying the number of alpha-particle hits per nucleus. These three tools were used to investigate cellular response as a function of time and distance from the Alpha-DaRT source in a two-dimensional (2D) culture system. To make the analysis more quantitative, we used a medium containing daughter radionuclides of ^224^Ra (“daughter medium”) at various dilutions to establish a correlation between the number of γH2AX foci and number of detected alpha-particle CR-39 etch pits, and employed a Monte-Carlo simulation to further relate this to the total number of nucleus hits and the absorbed dose. This combination of experimental methods allowed us to discuss the Alpha-DaRT-induced cell fate from the viewpoint of DDR and explore the potential usefulness of DDR as a predictor for the efficacy of Alpha-DaRT treatments.

## Results

### Daughter nuclides from the ^224^Ra Alpha-DaRT source do not adsorb to tumor cells in culture conditions

**Figure 2:**
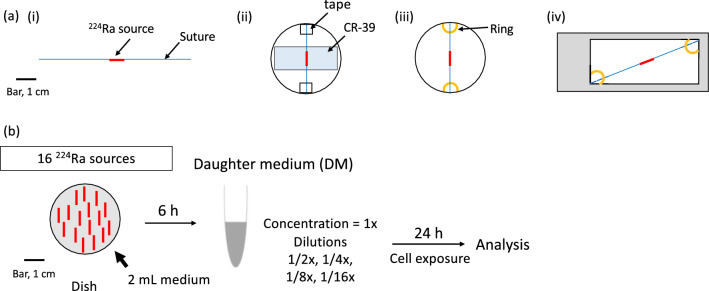
Experimental designs using the ^224^Ra source and CR-39 in this study. (**a**) Structure and positions of ^224^Ra source and CR-39, (i) Structure of the ^224^Ra source; (ii) Position of CR-39 in a plastic culture dish; (iii) Position of ^224^Ra source in a plastic dish; (iv) Position of ^224^Ra source in a slide chamber. Sutures were fixed by tape or plastic rings using grease. (**b**) Preparation of daughter medium (DM) from ^224^Ra sources. Sixteen ^224^Ra sources were incubated in 2 mL of growth medium for 6 h, the concentration of which was defined as 1 ×. Cells grown on the culture dishes were incubated in the indicated dilutions of DM for 24 h and used for various analyses.

Lead is known to bind to a variety of proteins and easily adsorbs to erythrocytes^27,28^. It may be possible that ^212^Pb also adsorbs to HeLa-Fucci cells, thereby affecting alpha particle distribution in the present culture conditions (see Fig. 2a). To test this possibility, we used CR-39, a plastic nuclear track detector^29^. A daughter medium (DM) containing daughter nuclides prepared from the ^224^Ra sources (Fig. 2b) was added onto the CR-39 plates on which HeLa cells were cultured or absent (Fig. 2a-ii without the ^224^Ra source). Given the short half-lives of ^220^Rn (55.6 s) and ^226^Po (0.145 s) and initial uniform distribution, it is unlikely that substantial portion of them is adsorbed to the HeLa cells before they decay. Thus, the majority of daughter nuclides influencing cells should be those after ^212^Pb (10.64 h). No statistically significant difference was detected in the etch pit density between the plates with and without the cells (Supplementary Fig. 1a,b). Furthermore, in the former case, no difference was detected between the two areas on the same CR-39 plate in the presence or absence of cells (Supplementary Fig. 1c,d). These results indicate that ^212^Pb derived from ^224^Ra is unlikely to significantly adsorb to HeLa cells. Thereafter, for the detection of alpha particles, the CR-39 was used without culturing cells on it.

### Distribution of alpha particles derived from daughter nuclides of the ^224^Ra source

**Figure 3:**
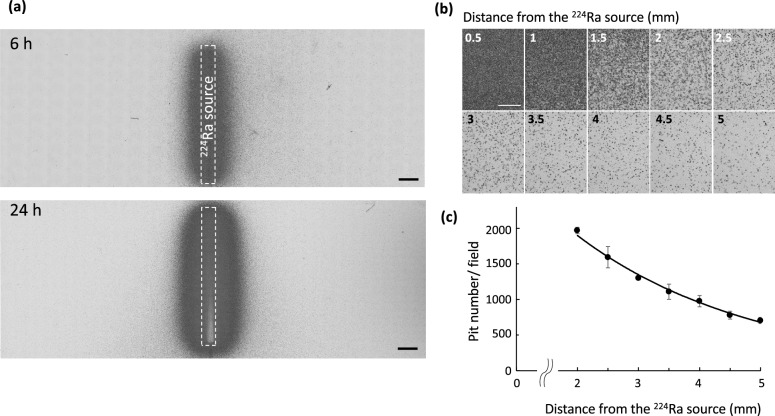
Distribution of alpha particles derived from the ^224^Ra Alpha-DaRT source. (**a**) Representative images of etch pits after source exposure. After exposure to the source for 6 or 24 h, the CR-39 was etched and photographed in low magnification. Bar, 1 mm. (**b**) High magnification images of etch pits at different distances from the source after 24 h of exposure. Bar, 100 μm. (**c**) Quantitative analysis of (**b**). Counting of the etch pits was only possible beyond 2 mm from the source and the data were plotted against the distance from the source. Data are represented as means ± SD of three independent areas.

To visualize the distribution of alpha particles, CR-39 was exposed to alpha particles derived from daughter nuclides of the ^224^Ra source either 6 h or 24 h after the start of exposure in a setup as shown in Fig. 2a-ii. Many etch pits were already formed in the area close to the source after 6 h (Fig. 3a, upper panel). Twenty-four hours after the start of irradiation, the high-density area expanded further (Fig. 3a, lower panel). For the 24-h exposure, the number of etch pits could not be counted within approximately 2 mm from the source due to overlaps of the pits. Outside of this range, it could be quantified, and the number/observation field (98,826 μm^2^) plotted against the distance from the source is shown in Fig. 3b,c. A representative image for 72 h exposure is shown in Supplementary Fig. 2. The expansion of the high-intensity area was very limited compared to 24 h and only slight diffusion outside of the dense area was observed even with the longer etching treatment (6 h) (see “Methods”). This is consistent with the exponential buildup timeline of ^212^Pb inside the medium, which stabilizes on its asymptotic form roughly 1 day after the introduction of the ^224^Ra source into the medium^14^.

### Distribution of DNA damage in HeLa-Fucci cells exposed to alpha particles from the ^224^Ra source

**Figure 4:**
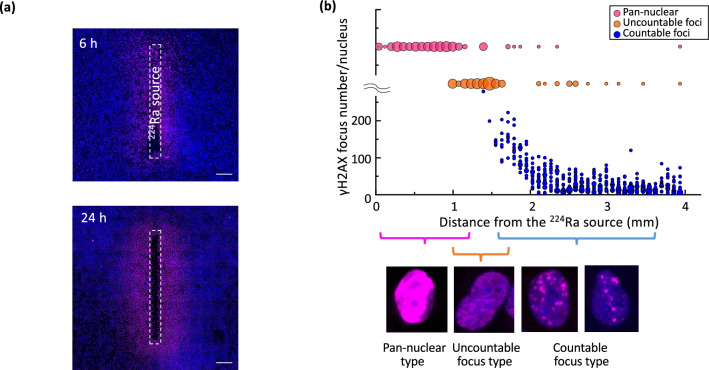
Distribution of DNA damage in cells exposed to alpha particles from the ^224^Ra source. (**a**) Fluorescence immunostaining of γH2AX after 6 h (upper panel) or 24 h of exposure (lower panel). Images of γH2AX (magenta) and nuclei (blue) are merged. Bar, 1 mm. (**b**) Quantitative analysis of γH2AX foci per nucleus. Staining patterns are divided into three foci types and shown as a bubble plot against distance from the source. The bubble size corresponds to the cell number. Magenta, orange, and blue bubbles show pan-nuclear, uncountable, and countable types, respectively. Lower panels show representative images of each foci type.

We next attempted to visualize DDR using the design shown in Fig. 2a-iv. For the detection of DNA damage, immunostaining for γH2AX, a very sensitive DNA double-strand break (DSB) marker, was employed^30^. Using low magnification (× 10), fluorescence images were obtained after 6 h or 24 h of exposure as shown in Fig. 4a. γH2AX stained in magenta was observed around the source, corresponding to the high etch pit density areas (Fig. 3a). After a 24-h exposure, the stained areas extended up to approximately 1–1.5 mm from the source, and beyond that distance density was remarkably reduced (Fig. 4a, lower panel). In high magnification images (× 100), the number of γH2AX foci per nucleus was quantified after 24 h of exposure. A large number of γH2AX foci were detected in the vicinity of the source, and a distance-dependent decrease in the intensity was detected (Fig. 4b). Within approximately 1 mm from the source, many “pan-nuclear type” cells (i.e., cells whose entire nucleus was uniformly stained with no ability to distinguish individual foci) were detected, that were considered no longer able to survive^31^. At approximately 1.5 mm from the source, the foci were visible but could not be counted due to overlaps (uncountable foci type). Beyond the line, a countable foci type appeared, and their number decreased in a distance-dependent manner (Fig. 4b).

### Temporo-spatial information on cell cycle kinetics in HeLa-Fucci cells exposed to alpha particles from the ^224^Ra source

**Figure 5:**
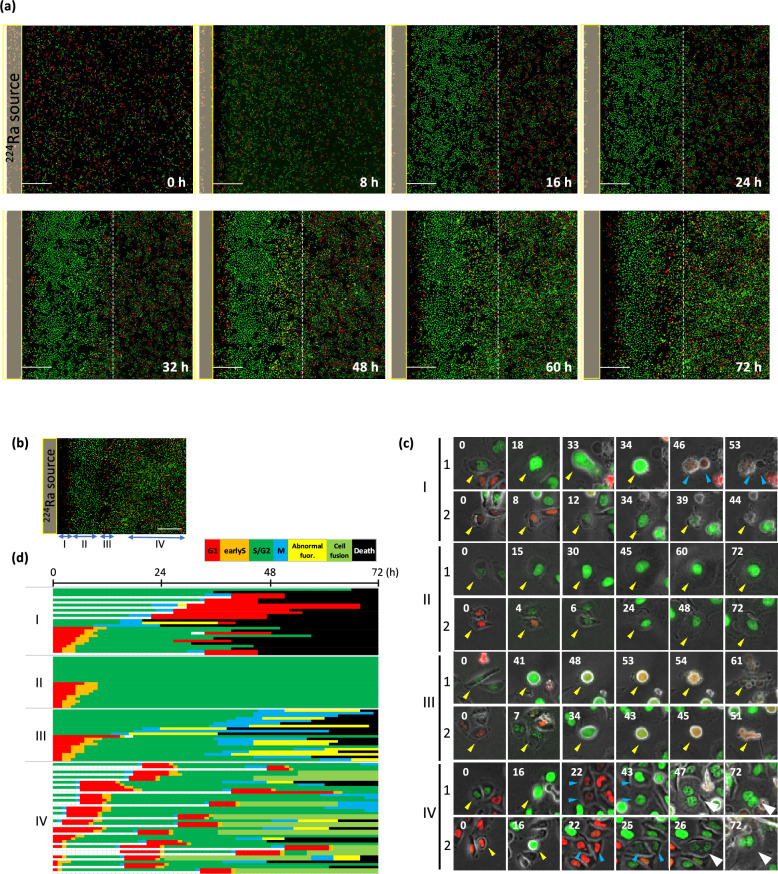
Time-lapse imaging of cell cycle kinetics in cells exposed to alpha particles from the ^224^Ra source. (**a**) Representative images of the same area observed over time after exposure. Dashed white line represents the outermost line of the first G2 arrest wave. Bar, 1 mm. (**b**) Four areas from the source (I-IV) showing distinct cell cycle kinetics on the image of 72 h in (**a**). Bar, 1 mm. (**c**) Representative images of each area as defined in (**b**). Cells exhibiting representative kinetics are shown, starting in the green or red phase by Fucci. Yellow and blue arrowheads represent before and after cell division, and white arrowheads indicate cells showing cell fusion. Time is shown in hours with the starting point at 0 h. (**d**) Pedigree analysis of Fucci fluorescence in each position as defined in (**b**). Ten cells belonging to each area starting at green or red phase were selected. Each cell was colored according to the length of fluorescence duration based on the data obtained from time-lapse imaging in (**c**). Red, G1; Green, S/G2/M.

In previous works we extensively studied G2 arrest kinetics using HeLa-Fucci cells and showed that the cells exhibited clear G2 arrest following X-irradiation, almost perfectly corresponding to the accumulation of green cells (G2 phase) by combining a time-lapse imaging technique with DNA content analysis by fluorescence activated cell sorter (FACS)^25,32^. Using the same system, we could obtain detailed information regarding temporo-spatial cell cycle kinetics during the exposure in this study. Before the exposure, red (G1 phase) and green (S/G2/M phases) fluorescence were evenly distributed in the observation field. Entering M-phase could be distinguished by nuclear envelope breakdown detectable by spread of Fucci fluorescence from the nucleus or round shape of the cell. Fluorescence images of cells grown on a plastic culture dish (Fig. 2a-iii) were acquired as shown in Fig. 5a up to 72 h of exposure. At 16 h of exposure, most cells located up to approximately 2.5 mm from the source (dotted white line in Fig. 5a) became green, showing that cells arrested at the G2 phase, and the intensity increased after 24 h of exposure, indicating that the G2 arrest continued. This was confirmed by the observation that a WEE1 inhibitor, functioning as a G2/M checkpoint inhibitor, substantially reduced the accumulation of green cells (Supplementary Fig. 3).

To analyze the cell cycle kinetics in detail at the single-cell level, we divided the cell areas into I to IV, depending on the distance from the source as shown in Fig. 5b. Pedigrees were also determined for each cell located in areas I–IV, starting from the green or red phase (Fig. 5c). In area I, cell death occurred at the following timings: G1 phase after mitosis (mitotic death) (Fig. 5c: I-1) and G2 phase before entering M-phase (interphase death) (Fig. 5c: I-2). In area II, G2 arrest persisted in most cells and no cases of mitotic or interphase death were observed up to 72 h after exposure initiation (Fig. 5c: II-1 and -2). In area III, the G2 arrest released earlier than in area II, and cell death occurred during mitosis. Notably, most cells exhibited remarkable elongation of mitosis, accompanied by abnormal Fucci fluorescence (yellow: green + red) during mitosis (Fig. 5c: III-1 and -2), eventually leading to cell death. The phenomenon observed in areas I-1 and III is known as checkpoint adaptation; namely, G2 arrest was released to enter M-phase carrying massive DNA damage, subsequently causing enhanced cell death^33^. This differs from the normal release from the G2 arrest after most of the DNA damage is repaired, leading to cell survival^33^. In area IV, the cell cycle progressed almost normally^24^ and cells divided up to 24 h (Fig. 5c: IV-1 and -2). Thereafter, G2-arrested cells gradually appeared with variations. Micronuclei, appearing when cells enter mitosis with DSBs^34,35^ (IV-2, 22 h) and cell fusion (IV-1 and 2, 72 h) were also observed.

### Spatial comparison in distribution of alpha particles and DDR in merged images

**Figure 6:**
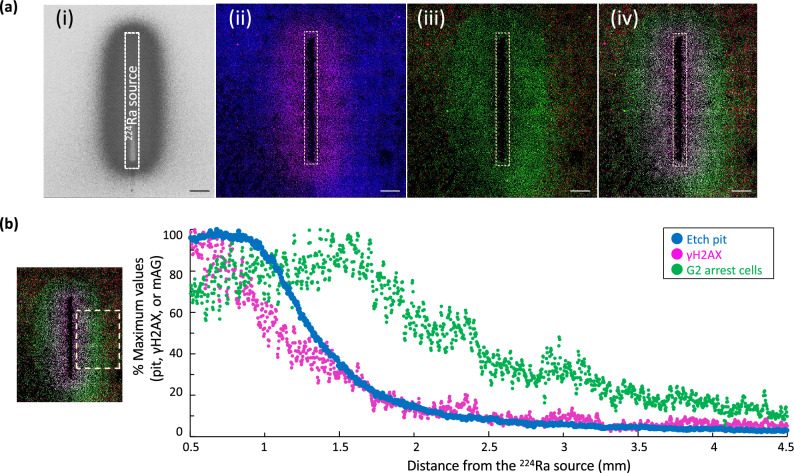
Merging of images representing distribution of alpha particle etch pits and DNA damage response. (**a**) Each representative image when exposed to the source for 24 h. a, Etch pits; b, γH2AX (magenta) and nuclei (blue); c, Fucci fluorescence; d, Merge. For acquisition of the Fucci fluorescence, cells grown on a slide chamber were used and the same cells on the chamber were immunostained for γH2AX. Bar, 1 mm. (**b**) Line profile analysis of (**a**). The area of the dashed square (left panel) was analyzed. Relative intensities were corrected with the respective maximum values as 100% and plotted against the distance from the source. Each dot represents the relative total fluorescence intensity within the scanning window area of 4.2 μm × 4 mm.

To integrate the spatial information regarding the DNA damage, G2 arrest, and alpha particle density, we attempted to merge the data obtained after the 24-h exposure. This revealed that the area irradiated by a high density of alpha particles almost coincided with that of cells with strong intensity for γH2AX visualized using low magnification (× 10) (Fig. 6a). Cells in this area mostly consisted of the pan-nuclear type and partly uncountable foci type in the γH2AX immunostaining patterns (Fig. 4b). Notably, the area of cells showing strong intensity of green Fucci fluorescence, namely, long G2 arrest, was much wider compared to two other areas, reaching up to approximately 2.5 mm from the source (Fig. 6a). The quantitative analysis by the Plot Profile confirmed the results (Fig. 6b). The time course of intensity of Fucci green fluorescence for cells in Fig. 5 is also shown in Supplementary Fig. 4.

Sham-irradiation controls are demonstrated in Supplementary Fig. 5. Using an exhausted ^224^Ra source (manufactured a year prior to the experiment), no essential changes were observed for γH2AX focus formation and cell cycle kinetics. Cells normally grew and almost became confluent after 72 h incubation with the sham-irradiation source.

### Effects of DM containing daughter nuclides on DDR

**Figure 7:**
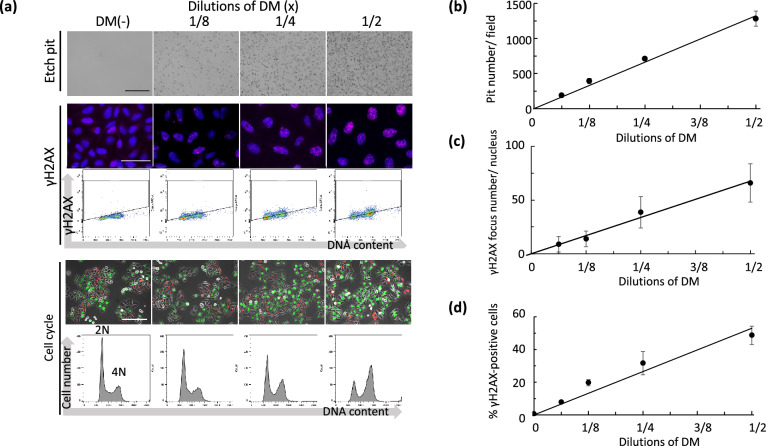
Effects of the DM on DNA damage response. (**a**) Representative images of each response by different concentrations of DM for 24 h of exposure. Upper panel, etch pits. Bar, 100 μm. Middle panel, fluorescence immunostaining of γH2AX (magenta) and nuclei (blue) (upper), and 2D flow cytometric analysis of DNA content and γH2AX expression (lower). Bar, 50 μm. Lower panel, Fucci fluorescence (upper) and flow cytometric analysis of DNA contents (lower). Bar, 200 μm. (**b**) Quantification of etch pits per field. Data are represented as means ± SD of five independent fields. r^2^ = 1.00. (**c**) Quantification of γH2AX focus number per nucleus. Data are represented as means ± SD. r^2^ = 0.99. N = 50 in each concentration. (**d**) Quantification of γH2AX intensity by flow cytometric analysis. Data are represented as means ± SD of three independent experiments. r^2^ = 0.98.

The experimental conditions shown in Fig. 2a gave us temporo-spatial information on the etch pit density around the Alpha-DaRT source; here, we relate this more quantitatively to DDR by employing results from the studies using the DM. The DM contained ^212^Pb with a long half-life and its daughter nuclides (^212^Bi and ^212^Po) emitting alpha particles, in secular equilibrium (as well as the beta emitter ^208^Tl). Figure 7a shows the images of etch pits and γH2AX staining, percent of γH2AX-positive cells, and cell cycle kinetics when cells were exposed to various dilutions of the DM for 24 h. A linear correlation was obtained between the number of etch pits and dilution factors (Fig. 7b). For γH2AX detection, countable foci type was detected at less than 1/2× the concentration and the foci number increased linearly as the concentrations increased (Fig. 7c). Likewise, a linear relationship was also obtained between the percent of γH2AX-positive cells and dilution factors as quantified by FACS (Fig. 7d). For cell cycle kinetics, it was confirmed visually that the fraction of G2-arrested cells with green fluorescence increased in a concentration-dependent manner, which was consistent with the results obtained from DNA content analysis (Fig. 7a, bottom panel). Taken together with the data of Figs. 3c and 4b, the etch pit density and DNA damage level at 2–3 mm from the source for 24 h corresponded to those given by the 1/2× concentration of DM for 24 h.

### Determination of cell surviving fractions after exposure to the DM

**Figure 8:**
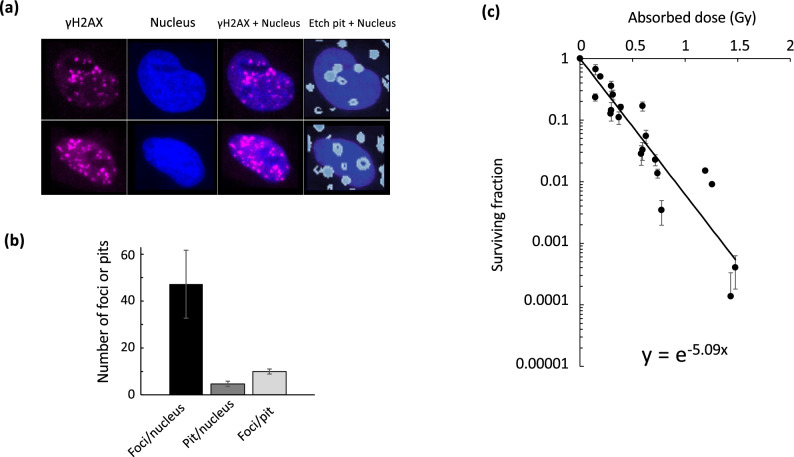
Estimation of cell survival curve exposed to the DM for 24 h. (**a**) Two representative images of γH2AX (magenta), nuclei (blue), and etch pits (light blue). CR-39 or cells grown on chamber slides were incubated in the 1/2× concentration of DM for 24 h. Etch pits or γH2AX foci together with nuclei were detected, and the images were merged at the same magnification. (**b**) Quantitative analysis of γH2AX foci number and etch pits per nucleus after exposure to the 1/2× DM for 24 h. The number of γH2AX foci per nucleus, etch pits per nucleus, and foci per pit were calculated based on the nuclear size after the 1/2× DM exposure. Data are represented as means ± SD of three independent experiments. (**c**) Cell survival curve for cells after exposure to varying concentrations of DM for 24 h. Cells were exposed to varying dilutions of DM for 24 h, and surviving fractions were obtained by colony formation assay. Surviving fractions were plotted against radiation doses estimated as shown in Appendix. Data are represented as means ± SD of at least three independent dishes. r^2^ = 0.87. Regression line: y = exp(− 5.09x).

The DM approach also allowed us to perform a colony formation assay to determine survival fractions after incubation with the DM. After cells were incubated with varying dilutions of DM for 24 h, surviving fractions were determined. Since the activities of the ^224^Ra sources varied depending on each distribution date from the supplier and the start times of the experiments, they were adjusted to the start of the experiments according to the half-life of ^224^Ra. Surviving fractions were plotted against etch pit numbers/nucleus. Each etch pit number was determined according to the linear relationship between etch pit numbers and dilution factors of the DM with adjustment. The shape of the nucleus could be clearly delineated in the fluorescent images and its average area was determined as well. After 24 h of exposure to the 1/2× DM, representative images for γH2AX staining and nucleus, and merge of nucleus, γH2AX staining, and etch pits on the CR-39 detector are shown in Fig. 8a. The average area of the nucleus, etch pits/nucleus, and γH2AX focus number/nucleus were 280 ± 39 μm^2^, 4.7 ± 1.1, and 47.2 ± 14.5, respectively (Fig. 8b). This situation may reconstitute the condition around the cells located in areas II–III as defined in Fig. 5b after 24 h of exposure. The cell survival curve was linear on a logarithmic scale, lacking a shoulder; this is typical for exposures to alpha particles, with LET values > 100 keV/μm (Supplementary Fig. 6). The surviving fraction was 0.0037 when the average etch pit number/nucleus was 4.7.

The Appendix as shown in Supplementary material describes a Monte-Carlo-based analysis, from which the number of alpha particles hitting the nucleus (including those not leaving detectable etch pits, and those moving with an upward momentum) at this irradiation level (24 h of exposure to the 1/2× DM) was 15.6 ± 3.3 (assuming the alpha emitters are uniformly distributed throughout the medium, including the cell nucleus inner volume). Furthermore, the absorbed dose from alpha particles was estimated as described in Appendix. Judging from the cell survival curve as a function of the number of etch pits per nucleus, the survival probability for 4.7 etch pits per nucleus was 3.75 × 10^−3^ when exposed to the ×1/2 DM for 24 h. This corresponded to a dose of 1.10 ± 0.23 Gy. The survival curve in Fig. 8c was redrawn by converting the abscissa from the pit number (Supplementary Fig. 6) to the absorbed dose, following the explanation in Appendix. The exponential fit showed that the mean lethal dose D_0_ (radiation dose reducing the surviving fraction to 1/e) was 0.20 ± 0.04 Gy, demonstrating essentially the same radiosensitivity as that of HeLa-S3 cells (D_0_ = 0.25 Gy) determined by exposure to alpha particles from ^241^Am^36^.

## Discussion

In this study, we succeeded in visualizing the time- and space-dependent distribution of alpha-particle nucleus hits, DNA damage, and cell-cycle dynamics after exposure to a ^224^Ra Alpha-DaRT source under specially-prepared in-vitro conditions. The use of a radionuclide “daughter medium” (DM) containing ^212^Pb and its daughters ^212^Bi, ^212^Po, and ^208^Tl, allowed us to demonstrate a linear relation between the number of γH2AX foci per nucleus and the density of detected alpha-particle etch pits in CR-39. Using a Monte-Carlo microdosimetry calculation, it further enabled us to estimate the total number of alpha particle hits to the nucleus, the absorbed dose and cell survival as a function of dose.

With respect to cell-cycle dynamics, up to 24 h after the start of exposure most cells exhibited similar G2 arrest kinetics in areas I–III (Fig. 5b), up to ~ 2.5 mm from the source. Thereafter, cells showed varying cell kinetics, depending on the cell positions relative to the source. Close to the source (area I, up to ~ 0.5 mm from the source), cells received a huge number of alpha particles, resulting in a pan-nuclear type of γH2AX staining up to 24 h (i.e., a fully γH2AX-stained nucleus). Given that the DNA damage with this staining pattern is no longer repaired^31^, cells in this area would be almost eradicated thereafter. Farther away from the source (areas II and III, at a distance of ~ 0.5–2.5 mm), the number of alpha-particle hits to nuclei gradually dropped. The γH2AX staining patterns changed as a function of distance in these areas, with the number of foci shifting from being uncountable to countable up to 24 h. Cells in area II showed extended G2 arrest, while cells in area III showed release of the G2 arrest and started to die at 48 h. The cells in area IV (> 2.5 mm from the source) were likely to proliferate at first, but gradually showed G2 arrest from 24 h. The first wave of G2 arrest occurring in areas I-III and the second wave later occurring in area IV could be clearly distinguished by 24 h or even 16 h of exposure. The border was not detectable by using the γH2AX staining pattern or the number of γH2AX foci per nucleus, suggesting that the Fucci system was somehow able to draw the line (i.e., display a threshold effect) by sensing the subtle difference of the DNA damage level inducing G2 arrest (Fig. 5a). The second wave of G2 arrest appeared in area IV at a later time (Supplementary Fig. 4). The range of area IV and the extent of cell death were not fully studied due to the limitations of the experimental conditions. Notably, G2 arrest extended to larger distances from the source compared to the γH2AX staining pattern.

In clinical settings, the optimal source arrangement is a hexagonal lattice, and equivalently, the unit cell can be defined as an equilateral triangle by source triplets^16^. In this geometry, the minimum dose is found around the center of gravity of each triangle (red circles in Supplementary Fig. 7). Thus far, regions affected by the ^224^Ra source in vivo were evaluated by necrosis 4–7 days after treatment^8,11^, where tumor tissues surrounding the source collapsed, and the precise tissue position was not maintained. From our findings, the first G2 arrest wave, having a high cell-death potential, was clearly delineated after 24 h of exposure where tumor tissues were still intact. This prompts us to consider whether the extent of the first G2-arrest wave into this region could be a potential predictor for the success of Alpha-DaRT treatments. Since it is conceivable that the size of necrosis, extent of fibrosis, tumor vessel density, and extent of hypoxia, etc., could affect the diffusion and leakage of the daughter nuclides, the above-described marker could also play an important role in preclinical studies to explore such effects. On the other hand, it is unclear how the second G2 arrest wave influences the effective kill range induced by ^224^Ra Alpha-DaRT sources in the current study. Including the effects of the second G2 arrest wave, we must await in vivo analysis.

Based on the Monte-Carlo simulation, we could estimate the radiosensitivity of HeLa-Fucci cells exposed to alpha particles from ^212^Bi and ^212^Po using several dilutions of the DM. From the number of detected etch pits per nucleus, the simulation allowed estimating the total number of nucleus hits, which is about 3 times larger, and further provided an estimate of the absorbed dose from alpha particles. The mean lethal dose D_0_ was found to be 0.20 ± 0.04 Gy (see Appendix), demonstrating essentially the same radiosensitivity, within error, as that of HeLa-S3 cells (D_0_ = 0.25 Gy) determined by exposure to alpha particles from ^241^Am with a dose rate of 0.3 Gy/min^36^. As shown in Fig. 8b, cells exposed to the 1/2× DM for 24 h exhibited 4.7 ± 1.1 etch pits on the CR-39, showing a surviving fraction of 3.75 × 10^−3^. The corresponding dose was estimated as 1.10 ± 0.23 Gy and the average dose rate was 7.6 × 10^−4^ Gy/min. It is interesting to note that alpha irradiation even in this extremely low dose rate induced a similar level of radiosensitivity as that induced by much higher dose rate of alpha irradiation. In general, surviving fractions gradually increase as dose rates decrease when cells are irradiated by low-LET radiation. This is called the “dose-rate effect” (DRE) and strongly depends on the extent of sublethal damage repair (SLDR)^1^. In the present study, despite the extremely low dose rate, the survival curve was purely exponential (i.e., linear on a logarithmic scale), similar to the survival curve obtained by Sasaki^36^, representing essentially no SLDR, eventually leading to no DRE.

For dose rates spanning the range 6.2 × 10^−3^ to 1.2 × 10^−2^ Gy/min, given by X-irradiation, HeLa-S3 cells exhibited rather lower surviving fractions compared to those at higher dose rates (~ 2.6 × 10^−2^ Gy/min)^37^. This is called the “inverse dose rate effect” (IDRE), occurring because cells accumulate in the G2 phase during the long irradiation period, where cells become radiosensitive^38^. In this study, even at a much lower dose rate (7.6 × 10^−4^ Gy/min), exposure to alpha particles caused significant accumulation of cells in the G2 phase (Figs. 5, 6, 7, 8). Given that alpha-irradiation still caused higher radiosensitivity in the G2 phase in HeLa-S3 cells to some extent^36^, the IDRE might also occur here even after alpha-irradiation. Alpha-particle-induced bystander effects at low doses, influenced by secreted factors or via gap junction intercellular communication from irradiated cells to unirradiated cells, could play an important role in the present experimental conditions as well^39–41^. For instance, cells without focus formation observed after 24 h exposure to the 1/8 DM (Fig. 7a) may have been subject to bystander effects. Taken together, such varying biological mechanisms occurring even under extremely low-dose-rate irradiation could not be ruled out to explain the high radiosensitivity comparable to that in high-dose-rate irradiation. Further study is required to pursue which factors are significant.

One might argue that our in vitro experimental conditions do not reflect in vivo conditions. Indeed, the behavior of radionuclide diffusion with higher diffusion coefficients in the liquid compared to tumor tissue, and with possible enhancement by convective currents in the medium resulting from thermal gradients or low vibrations, manifests a slow spatial fall-off compared to that observed in mouse tumors^8^, or predicted by the tumor dose model^14–16^. However, the qualitative behavior of cell response down the alpha dose gradient could be expected to occur also inside tumors.

In conclusion, we characterized DDR in the 2D-culture conditions for the first time, qualitatively mimicking the Alpha-DaRT procedure and revealed the detailed cell cycle kinetics possibly leading to radiosensitization. Furthermore, the first G2 arrest area indicated by the Fucci system could be a potential early marker for identifying the effective therapeutic range induced by ^224^Ra Alpha-DaRT sources. Detailed analyses in the in vivo experimental conditions are required as a next step to validate in vitro findings.

## Methods

### Cell line and culture conditions

HeLa cells expressing Fucci (SA) (HeLa-Fucci) were provided by the RIKEN BRC through the National Bio-Resource Project MEXT, Japan. p53 is non-functional due to HPV infection. Cells were maintained in DMEM (Sigma-Aldrich, St. Louis, MO, USA) containing a high concentration of glucose (4500 mg/L) with 100 units/mL penicillin and 100 µg/mL streptomycin supplemented with 10% fetal bovine serum, at 37 °C in a 5% CO_2_ humidified atmosphere.

### ^224^Ra Alpha-DaRT source setting

The Alpha-DaRT source was fixed with a biocompatible suture (Fig. 2a-i) and loaded inside a rigid needle of the applicator (Alpha-Tau Medical, Jerusalem, Israel). The space between the source and inside of the needle was sealed with glycerin to avoid the release of ^220^Rn prior to source insertion and provide viscous friction to hold the source inside the needle. The experimental source was 0.7 mm in diameter and 7 mm long. The source with a suture was set as shown in Fig. 2a. The sources with sutures were manufactured and distributed by Alpha-Tau Medical (Jerusalem, Israel) so that the activities were approximately 7.4 × 10^4^ Bq/source at the indicated date of the experiments. The activity of each source was measured using an alpha spectrometer to determine its ^224^Ra activity and ^220^Rn release rate (desorption probability)^14^ before shipping from the supplier.

### Detection of alpha particles

Alpha particles were detected using a plastic nuclear track detector CR-39 (BARYOTRAK-P: Nagase Landauer, Tsukuba, Japan). The source, CR-39, and the daughter medium (DM) prepared from ^224^Ra sources as described below, were set up as shown in Fig. 2a,b. After washing with water, the radiation-exposed CR-39 was etched in a 7 M NaOH solution at a constant temperature of 70 °C for 4 h, creating conical etch pits. The thickness of the etched layer was evaluated by measuring the thickness of 14 CR-39 plates before and after etching, using a screw micrometer (QuantuMike series 293, Mitutoyo, Kanagawa, Japan). Measurements were done at three different sites per plate, yielding 6.9 ± 3.7 μm for the removed layer. After etching, a BIOREVO BZ-9000 fluorescence microscope (KEYENCE, Osaka, Japan) was used to observe damage traces formed on CR-39 by incident alpha particles and bright field images were obtained. The number of etch pits was measured and quantified by ImageJ software (available from a website at http://rsbweb.nih.gov/ij/).

### Preparation of DM

To prepare the DM, sixteen ^224^Ra Alpha-DaRT sources were placed in a 35 mm dish and 2 mL of medium was added for complete immersion. The DM was collected in tubes after incubation for 6 h and diluted to the indicated concentrations, and the cells were then incubated in the DM for 24 h (Fig. 2b). Exposed cells in the DM were used for each experiment.

### Fluorescence immunostaining

Cells grown on Lab-Tek Chamber Slides (Nunc, Rochester, NY) for 24 h were exposed to the source or DM at each concentration for 24 h. Cells were then fixed using 4% paraformaldehyde in PBS for 20 min and then permeabilized in PBS containing 0.1% Triton-X-100 (PBS-T) at room temperature for 30 min. For detection of DSBs, cells were incubated at room temperature with an antibody against phosphorylated histone H2A.X (Ser 139) (1:500) (Cell Signaling, Danvers, MA) for 1 h after blocking in 10% normal goat serum (Invitrogen, Waltham, MA) for 30 min. After washing in PBS-T, cells were incubated for 30 min at room temperature with Alexa Fluor 647-conjugated goat anti-rabbit IgG (H + L) cross-adsorbed secondary antibody (1:500) (Invitrogen) containing 10 μg/mL Hoechst 33342 (Invitrogen) for 30 min and mounted with ProLong Gold Antifade Reagent (Life Technologies, Carlsbad, CA). Images were obtained using a BIOREVO BZ-9000 fluorescence microscope.

### Flow cytometric analysis

After the DM treatment at the indicated concentrations for 24 h, cells were washed three times to remove the daughter nuclides, trypsinized, and centrifuged. Cell pellets were washed in PBS and fixed using 4% paraformaldehyde in PBS for 20 min and then permeabilized with PBS-T for 30 min. To detect DSBs, staining was performed in the same manner as fluorescence immunostaining as described above. Finally, single-cell suspensions were filtered through a nylon mesh. Each sample was analyzed using a FACS Canto II (BD Bioscience, Franklin Lakes, NJ) with FlowJo software. All experiments were performed at least three times.

### Time-lapse imaging and pedigree analysis

Time-lapse images were acquired at a 1 h interval for 96 h on a BIOREVO BZ-9000 fluorescence microscope immediately after source setting. During imaging, cells grown on a plastic dish were held in an incubation chamber at 37 °C in a humidified atmosphere containing 95% air and 5% CO_2_ (Tokai Hit, Fujinomiya, Japan). For pedigree analysis, cells in each area were randomly selected from the acquired images. Pedigrees for each cell were established for a period of 72 h after the start of observation. The pedigrees were rendered as red (G1 phase), green (S/G2 phases), blue (mitosis), or black (cell death). Although cells within the S/G2/M phases similarly emitted green fluorescence, those in M phase could be distinguished morphologically.

### Line profile analysis

Density of etch pits, intensity of γH2AX staining, and Fucci green fluorescence were converted to grey level values and quantitated using a Plot Profile (ImageJ software) by scanning the areas of 4.5 × 5.7 mm^2^ for etch pits and 4.5 × 4.0 mm^2^ for γH2AX staining/Fucci green fluorescence with a window width of 4.2 μm. Each numerical value was corrected with the highest value as 100%.

### Colony formation assay

After 24 h incubation in varying dilutions of DM, cells were washed three times to remove the daughter nuclides and the appropriate number of cells following trypsinization were plated on cell culture plates. After incubation for approximately 8 days, the colonies were fixed using 4% paraformaldehyde and then stained with crystal violet. Colonies containing more than 50 cells were scored, and survival fractions were determined. The standard activity was basically 7.4 × 10^4^ Bq/source, but there were slight differences in activities depending on the distributed sources and start time of the experiments. Considering the half-life of ^224^Ra, radioactivities were corrected to the start of the source incubation for the DM preparation. Taking advantage of the linear relationship between dilutions of the DM and etch pit density, surviving fractions were plotted against the respective etch pit number/nucleus. The pit numbers detected by CR-39 per nucleus served as the starting point for a Monte Carlo simulation (described in detail in Appendix) which allowed to estimate the *total* number of corresponding hits to the nucleus. As detailed in Appendix, the total number of hits was ~ 3 times higher than the number of detected etch pits per nucleus. This is because only ~ 50–60% of the alpha particles hitting the CR-39 leave detectable pits, and in addition, there are alpha particles that either stop inside the nucleus without reaching the CR-39 or pass through the nucleus with an upward momentum. Scoring the energy deposited inside the simulated nucleus by each alpha-particle hit, the simulation further allowed to estimate the absorbed dose from alpha particles (the low-LET dose was estimated as well and found to have a negligible effect on cell survival). The relation between the detected number of etch pits and calculated absorbed dose allowed expressing cell survival as a function of the latter.

### Statistical analysis

A two-tailed *t*-test was performed. P-values < 0.05 were considered statistically significant.

### Supplementary Information

Supplementary Information.

Supplementary Figures.

## Data Availability

The datasets generated during and/or analyzed during the current study are available from the corresponding author on reasonable request.
