# Supplementary material for: DNA damage response in a 2D-culture model by diffusing alpha-emitters radiation therapy (Alpha-DaRT)
Source: Sci Rep. 2024 May 20;14:11468. doi: 10.1038/s41598-024-62071-6 (PMC11106084; doi:10.1038/s41598-024-62071-6)
Supplement: Supplementary file 1 — Supplementary Information. [file 41598_2024_62071_MOESM1_ESM.pdf]

Appendix: Estimating the mean number of hits to the cell nucleus and the absorbed dose from the density of recorded CR-39 etch pits

1. Estimating the fraction of alpha particles hitting CR-39 which create detectable etch pits

Only a fraction of the alpha particles hitting the CR-39 film create detectable etch pits. To create detectable pits, two conditions must be met: (1) the angle of the alpha particle track with respect to the CR-39 surface must be larger than an energy-dependent critical angle; (2) the penetration depth of the track into the CR-39 film must be larger than the thickness of the etched layer.

The dependence of the critical angle on the energy of the alpha particle as it hits the CR-39 surface is commonly parametrized through a sensitivity function ("V function") which depends on the residual range R' of the alpha particle in CR-39:

$$\psi_c(R') = sin^{-1}\big(1/V(R')\big) \quad (1)$$

There are several published parametrization forms of $V(R')$. Here we use a form by Hermsdorf [1]:

$$V(R') = 1 + \frac{a_1}{(R'+a_2)^{b_1}} \cdot ln(R' + a_3) \cdot \left(1 - e^{-R'/a_4}\right) + R'/a_5 \quad (2)$$

with $a_1 = 390\,\mu m$, $a_2 = 2\,\mu\text{m}$, $a_3 = 1\,\mu\text{m}$, $a_4 = 5\,\mu\text{m}$, $a_5 = 80\,\mu\text{m}$, $b_1 = 2.35$.

The calculation of the fraction of detected alpha particle hits was done separately for the three relevant alpha particle energies: 6.051 MeV (^{212}Bi, intensity 25.13%), 6.090 MeV (^{212}Bi, 9.75%), 8.785 MeV (^{212}Po, 100%). For each alpha particle energy, we performed the following steps, using an in-house MATLAB code:

1. The contiuous-slowing-down-approximation (CSDA) range in water, $R_{CSDA}(\mathrm{H_2O})$, was interpolated on tabulated NIST data (from the NIST ASTAR online database).

2. 10^8 alpha particle tracks were sampled from isotropic sources (emitting into a solid angle of 4π) with an initial height above the CR-39 plane z_0 sampled from a uniform distribution between 0 and $R_{CSDA}(H_2O)$.
3. For each alpha particle reaching $z = 0$ we calculated the residual energy E' (after traversing a path length $r = z_0/\cos\theta$, with θ being the emission angle relative to the negative z axis ($0 \leq \theta \leq \pi/2$). The residual range R' in CR-39 corresponding to the residual energy E' was found by interpolating on tabulated data from SRIM 2013 [2]. $V(R')$ was calculated following Equation (2) and the corresponding critical angle $\psi_c(R')$ was calculated using Equation (1).
4. An alpha particle was considered to be detected if the angle of its track relative to the CR-39 surface was larger than the critical angle, and if the depth of its penetration into the CR-39 film, $z_{depth} = R'\cos\theta$ was larger than the thickness of the etched layer (6.9 μm).

Table 1 shows the fraction of alpha particle hits detected for each energy. The errorbars correspond to varying the layer thickness by ±3.7 μm.

Table 1 Fraction of alpha particle hits detected

Alpha energy (MeV)	Fraction of detected hits
6.051	0.547 ± 0.108
6.090	0.548 ± 0.108
8.785	0.613 ± 0.060

2. Estimating the number of alpha decays per unit volume

Analytical derivation and Monte Carlo calculations show that for a uniform

distribution of mono-energetic alpha emitters in the medium (assumed here to be water), the relation between the number of alpha particle hits per unit area on the CR-39 surface (σ_{hit}) and the number density of alpha decays in the medium (n_{decays}) is:

$$n_{decays} = \frac{4}{R}\sigma_{hit} \tag{3}$$

where R is the CSDA range in water (with mean value of 50.47 μm for ^{212}Bi and 91.32 μm for ^{212}Po). Denoting by σ_{det} the number of detected hits per unit area, and by η the fraction of detected alpha particle hits ($\sigma_{det} = \eta\sigma_{hit}$) we have:

$$\sigma_{det}(tot) = \sigma_{det}(Bi) + \sigma_{det}(Po) = \eta_{Bi}\sigma_{hit}(Bi) + \eta_{Po}\sigma_{hit}(Po) \tag{4}$$

The ratio between the number density of ^{212}Po alpha decays ($n_{decays}(Po)$) to the number density of ^{212}Bi alpha decays ($n_{decays}(Bi\ \alpha)$) is 64/36. Using Equation (3):

$$\sigma_{det}(tot) = \frac{1}{4}n_{decays}(Bi\ \alpha)\left(\eta_{Bi}R_{Bi} + \frac{64}{36}\eta_{Po}R_{Po}\right) \tag{5}$$

The measured cell nucleus area is 280 ± 39 μm^2 and the number of detected etch pits per nucleus for the highest dose level (dilution 1/2×) is 4.7 ± 1.1. The density of detected pits is therefore: $\sigma_{det}(tot) = 0.0168 \pm 0.0032$ pits/μm^2. Inserting numerical values into Equation (5) gives: $n_{decays}(Bi\ \alpha) = (5.32 \pm 1.11) \cdot 10^{-4}$ decays/μm^3 and $n_{decays}(Po) = (9.46 \pm 1.98) \cdot 10^{-4}$ decays/μm^3.

3. Estimating the number of alpha hits per nucleus

The number of alpha hits per nucleus was estimated in a second in-house Monte Carlo simulation (implemented in MATLAB) as follows. The nucleus was simulated as an oblate spheroid with a horizontal radius $a = 9.44 \pm 0.66$ μm (corresponding to a

cross section area of 280 ± 39 μm^2) and a vertical radius $c = 5 \pm 1$ μm, with its center at $z = c$. Alpha particle tracks were generated randomly in a cylindrical domain surrounding the nucleus with a radius $R_{dom} = a + R$ and height $H_{dom} = 2c + R$, where R was set separately to the alpha particle CSDA range of ^{212}Bi and ^{212}Po. The alpha particle emission points were sampled uniformly throughout the cylindrical domain (including inside the nucleus) with isotropic directions. The calculation was performed separately for ^{212}Bi and ^{212}Po, counting in each case the number of straight tracks crossing the nucleus surface at least once. The ratio between the number of nucleus hits and number density of alpha decays (number of simulated decays divided by the domain volume) is given in Table 2. The uncertainties were estimated by sampling the spheroid radii randomly from normal distributions with their respective standard deviations.

Table 2 Ratio between the number of nucleus hits and number of decays per μm^3

Isotope	Number of nucleus hits/number of decays per unit volume (hits/decay per μm^3)
^{212}Bi	$(7.82 \pm 1.35) \cdot 10^3$
^{212}Po	$(12.05 \pm 2.04) \cdot 10^3$

Combining the values in Table 2 and estimated number of ^{212}Bi and ^{212}Po alpha decays given above, with their respective uncertainties, yields the estimated number of alpha particle hits to the nucleus for the highest dose (DM with dilution 1/2×):

$$N_{hits} = 15.6 \pm 3.3 \quad (6)$$

4. Estimating the absorbed dose

The MC simulation employed for estimating the mean number of alpha hits to the nucleus was further used to calculate the absorbed dose. For tracks crossing the nucleus surface at least once, we scored the energy deposited in the nucleus E_{dep} using dE/dX data taken from the NIST ASTAR online database (https://physics.nist.gov/PhysRefData/Star/Text/ASTAR.html). The specific energy for the *i*-th track was calculated as $z_i = E_{dep}^i / M_{nuc}$, where M_{nuc} is the nucleus mass (calculated assuming a medium density $\rho = 1$ g/cm^3). The total number of tracks for ^{212}Bi and ^{212}Po alpha decays was determined from the number of decays per unit volume corresponding to the most active DM (dilution ×1/2): $n_{decays}(Bi\ \alpha) = (5.32 \pm 1.11) \cdot 10^{-4}$ decays/μm^3 and $n_{decays}(Po) = (9.46 \pm 1.98) \cdot 10^{-4}$ decays/μm^3, and the volume of the cylindrical domain surrounding the nucleus (described above). This allowed scoring the total energy deposited in the nucleus $z = \sum z_i$ for a run simulating the real experiment. The process was repeated 10^4 times to produce the statistical distribution of the total specific energy $f(z)$. The absorbed alpha dose was then taken as average value of the distribution of the total specific energy, $D_\alpha = \bar{z} = \int z f(z)\, dz$. Using this methodology, for the most active DM (dilution 1/2×) we estimated an alpha dose of 1.10 ± 0.23 Gy.

The absorbed beta dose was grossly estimated as follows. For an infinite medium with a uniform concentration of ^{212}Pb with its beta-emitting daughters ^{212}Bi and ^{208}Tl in secular equilibrium:

$$D_\beta(inf) = \frac{1}{\rho}\left(n_{decays}(Pb)\bar{E}_\beta(Pb) + n_{decays}(Bi,\beta)\bar{E}_\beta(Bi) + n_{decays}(Tl)\bar{E}_\beta(Tl)\right) \quad (7)$$

where $n_{decays}(Pb)$ and $n_{decays}(Tl)$ are the respective number of decays of ^{212}Pb

and ^{208}Tl per unit volume, $n_{decays}(Bi,\beta)$ is the number of beta decays of ^{212}Bi per unit volume, and $\bar{E}_\beta(Pb) = 101$ keV, $\bar{E}_\beta(Bi) = 771$ keV, and $\bar{E}_\beta(Tl) = 560$ keV are the average energies of the beta electrons emitted by ^{212}Pb, ^{212}Bi and ^{208}Tl, respectively (taken from BNL's Nudat3 online database, https://www.nndc.bnl.gov/nudat3/). The individual number of decays are all related to the total number of decays of ^{212}Bi, $n_{decays}(Bi,tot) = 1/0.36\, n_{decays}(Bi,\alpha)$:

$$n_{decays}(Pb) = n_{decays}(Bi,tot) \quad (8)$$

$$n_{decays}(Bi,\beta) = 0.64\, n_{decays}(Bi,tot) \quad (9)$$

$$n_{decays}(Tl) = 0.36\, n_{decays}(Bi,tot) \quad (10)$$

For the most active DM (with $n_{decays}(Bi\ \alpha) = (5.32 \pm 1.11) \cdot 10^{-4}$ decays/μm^3) the "infinite medium" beta dose was calculated to be 0.19 ± 0.04 Gy. On the boundary between two infinite media, one with a uniform density of beta emitters and one with zero activity, the beta dose is precisely half of that in an infinite medium. As a very rough estimate, we adopted this value for the absorbed beta dose experienced by the cell, giving $D_\beta(cells) \approx 0.09$ Gy. Considering any reasonable value of RBE for alpha particles, and an alpha particle dose of 1.1 Gy, the relative beta contribution to cell survival can be expected to be at most on the few % level.

References

1. Hermsdorf, D. Evaluation of the sensitivity function V for registration of α-particles in PADC CR-39 solid state nuclear track detector material. *Radiat Meas* **44**, 283-288 (2009). https://doi.org:https://doi.org/10.1016/j.radmeas.2009.03.02
2. James F. Ziegler, M. D. Z. & J.P. Biersack. SRIM – The stopping and range of ions in matter (2010). *Nucl Instrum Methods Phys Res B* **268**, 1818-1823 (2010). https://doi.org:https://doi.org/10.1016/j.nimb.2010.02.091
