# Supplementary material for: DNA damage response in a 2D-culture model by diffusing alpha-emitters radiation therapy (Alpha-DaRT)
Source: Sci Rep. 2024 May 20;14:11468. doi: 10.1038/s41598-024-62071-6 (PMC11106084; doi:10.1038/s41598-024-62071-6)
Supplement: Supplementary file 2 — Supplementary Figures. [file 41598_2024_62071_MOESM2_ESM.pdf]

Supplementary Information

DNA damage response in a 2D-culture model by diffusing alpha-emitters radiation therapy (Alpha-DaRT)

Hitomi Nojima[1] Atsushi Kaida[1], Yusuke Matsuya[2,3], Motohiro Uo[4], Ryo-ichi Yoshimura[5], Lior Arazi[6], Masahiko Miura[1]*

1 Department of Dental Radiology and Radiation Oncology, Graduate School of Medical and Dental Sciences, Tokyo Medical and Dental University
2 Nuclear Science and Engineering Center, Japan Atomic Energy Agency
3 Faculty of Health Sciences, Hokkaido University
4 Department of Advanced Biomaterials, Graduate School of Medical and Dental Sciences, Tokyo Medical and Dental University
5 Department of Radiation Therapeutics and Oncology, Graduate School of Medical and Dental Sciences, Tokyo Medical and Dental University
6 Unit of Nuclear Engineering, Faculty of Engineering Sciences, Ben-Gurion University of the Negev

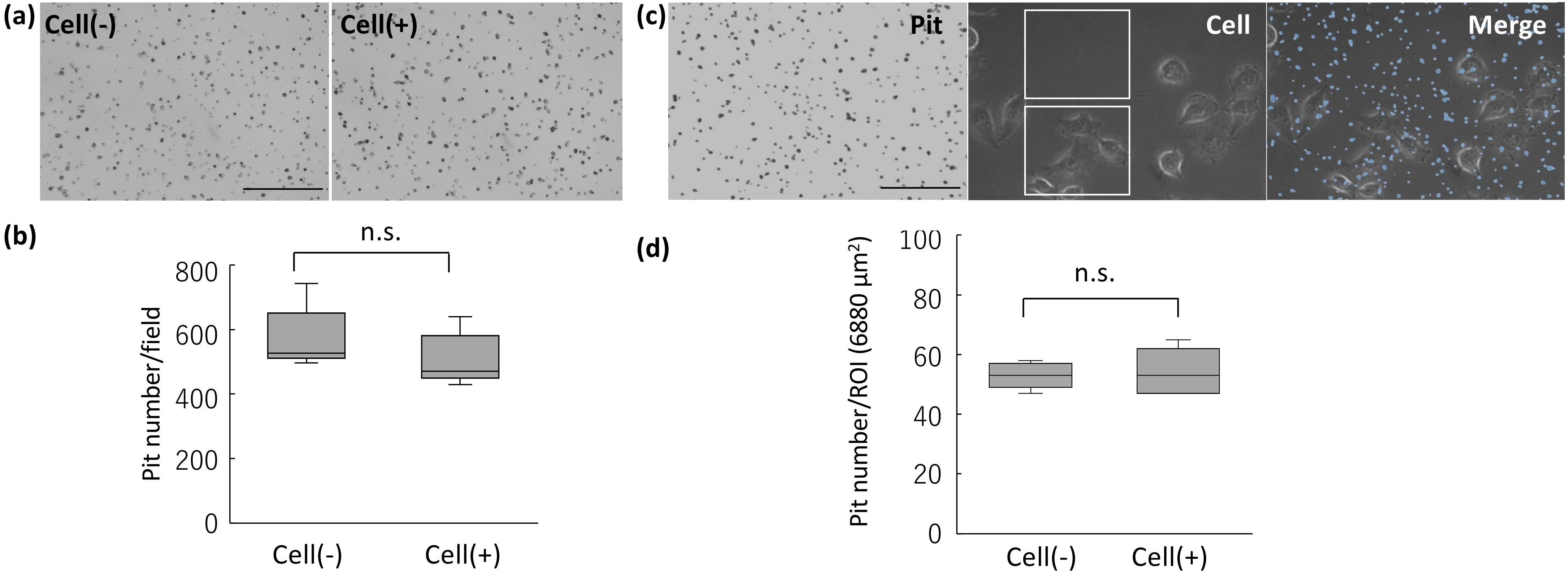

Supplementary Figure 1 Distribution of alpha particles on the CR-39 in the presence or absence of cells on the CR-39 plate.(a)Representative images of etch pits on the cell-present or -absent CR-39 plates. Bar, 100 μm. (b) Quantitative analysis of the etch pit density. The same size of ROI was set on two different CR-39 plates, cell-grown or not grown. Data are represented as box-whisker plots showing outliers, distribution intervals, 25–75% interquartile range (box), and median of 5 independent fields. A two-tailed t-test: ns, not significant. (c) Representative images of etch pits on the cell-present or -absent areas in the same cell-grown-CR-39 plate. Bar, 100 μm. (d) Quantitative analysis of the number of etch pits per field. The same size of ROI was set on the cell-present or -absent areas in the same cell-grown-CR-39 plate. Data are represented as box-whisker plots showing outliers, distribution intervals, 25–75% interquartile range (box), and median of 7 independent areas. A two-tailed t-test: ns, not significant. CR-39 plates were exposed to the daughter medium (DM) prepared from 16 ^{224}Ra sources in each experiment.

24 h exposure
4 h etching

72 h exposure
6 h etching

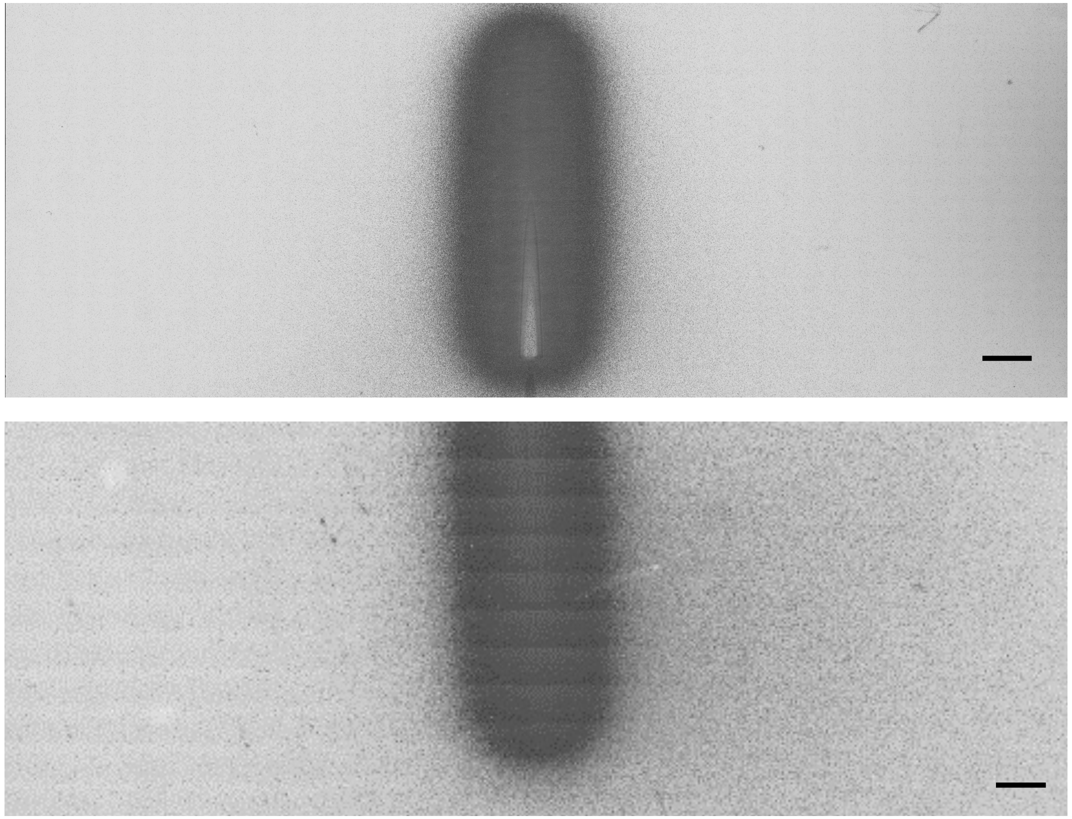

Supplementary Figure 2. **Distribution of pits created as a consequence of irradiation by alpha particles emitted by daughter nuclides that diffuse from the ^{224}Ra source**. A representative image of etch pits after source exposure for 72 h. The exposed CR-39 was etched and photographed in the same condition as Fig. 1a. Bar, 1 mm

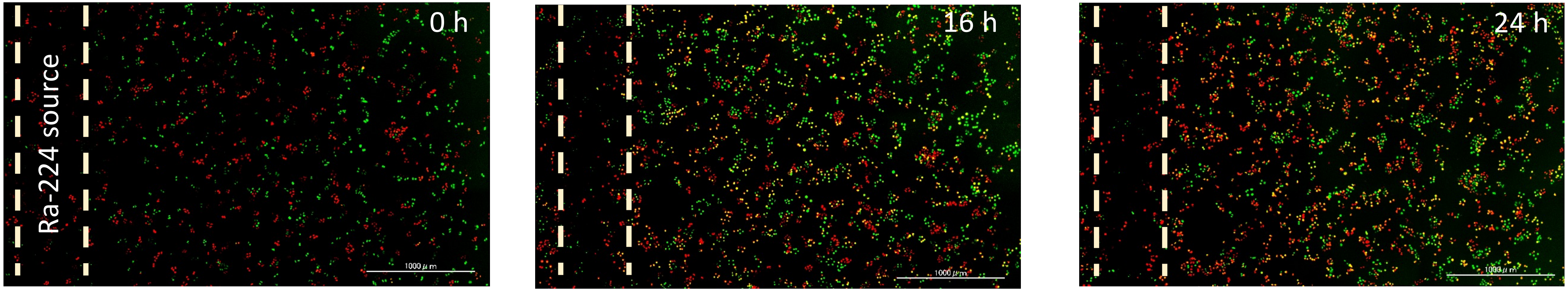

Supplementary Figure 3 WEE1 inhibitor substantially reduced the accumulation of green cells during exposure with ^{224}Ra source. Time-lapse images were acquired during exposure to the ^{224}Ra source in the presence of WEE1 inhibitor, MK-1775 (0.5μM). Bar, 1 mm.

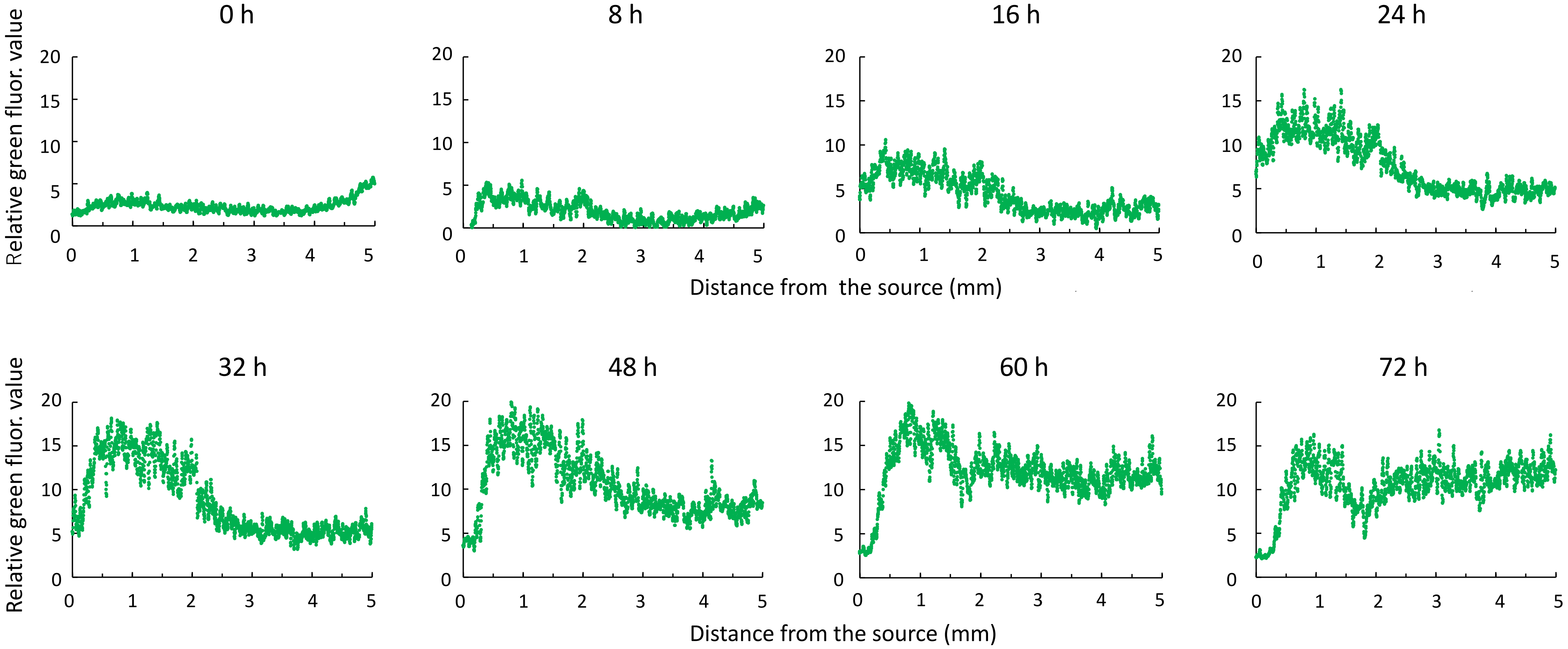

Supplementary Figure 4 Time course of intensity of Fucci green fluorescence for cells in Fig. 5. Line profile analysis was performed for Fucci green intensity in Fig. 5a at the indicated times. Relative intensities of Fucci green fluorescence were plotted against the distance from the source. Each dot represents relative total fluorescence intensity within the scanning window area of 4.2 µm × 4 mm.

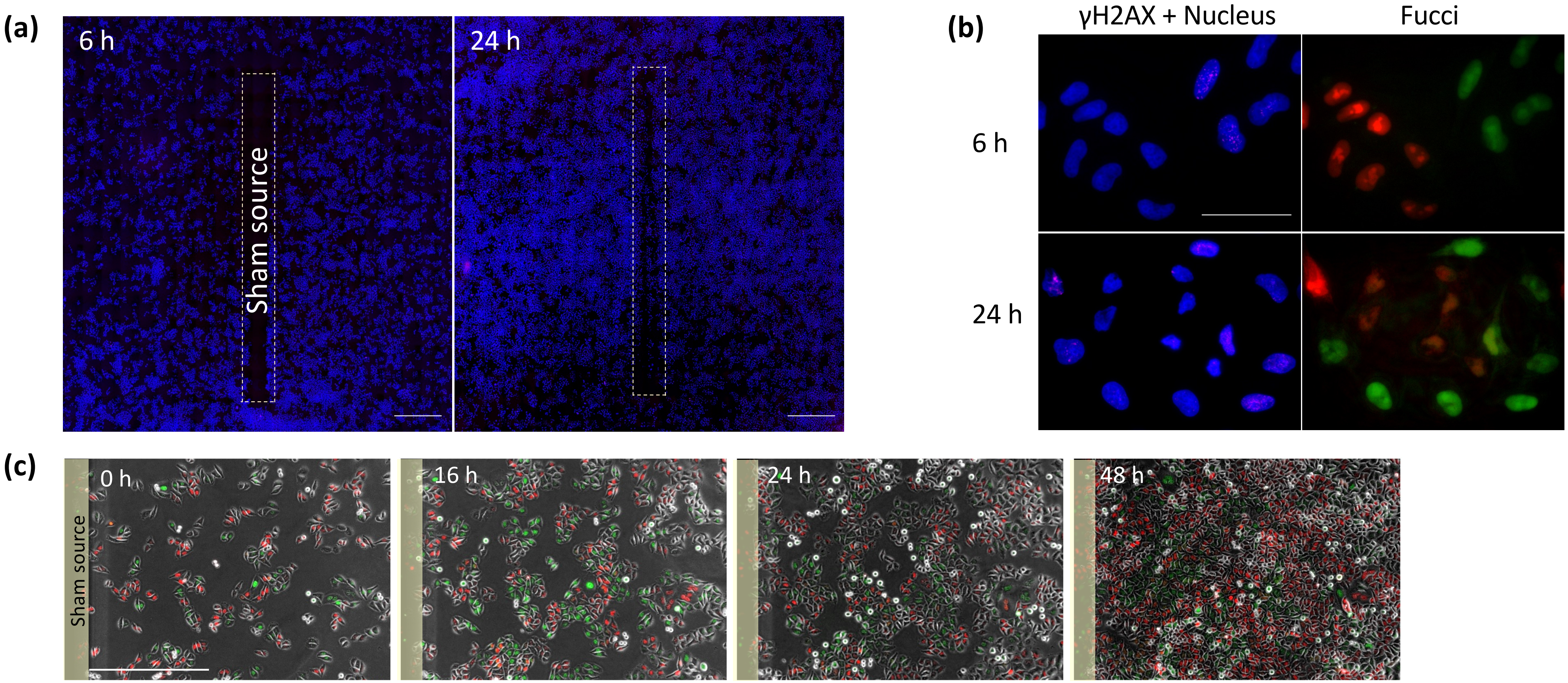

Supplementary Figure 5 DNA damage response after the sham-irradiation (a) Low-magnification fluorescence images at the indicated times after the sham-irradiation. Images of γH2AX (magenta) and nuclei (blue) are merged (a) Bar, 1 mm. (b) High-magnification fluorescence images of cells next to the sham source at the indicated times after the sham source exposure. Images of γH2AX and nuclei (left panel) or mAG (green) and mKO2 (red) (right panel) are merged. Bar, 50 µm. (c) Representative images of the same area observed over time after the sham source exposure. Bar, 500 µm. The sham-irradiation source is an exhausted ^{224}Ra source that was manufactured a year prior to the experiment.

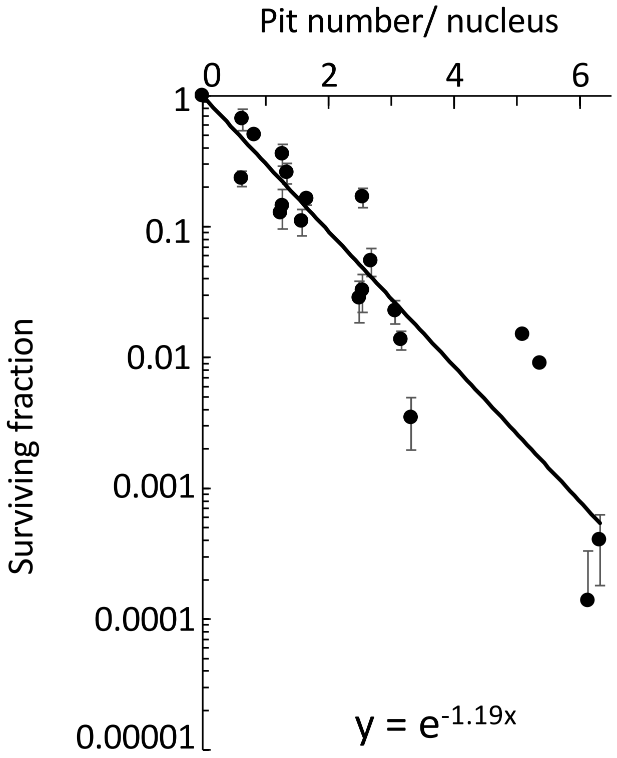

Supplementary Figure 6 Cell survival curve for cells after exposure to varying concentrations of DM for 24 h. Cells were exposed to varying dilutions of DM for 24 h, and surviving fractions were obtained by colony formation assay. Surviving fractions were plotted against the number of etch pits per nucleus corrected according to the estimated activities immediately before the DM preparation and linear relationship with the etch pit number. Data are represented as means ± SD of at least three independent dishes. $r^2 = 0.87$. Regression line: y = exp(-1.19x)

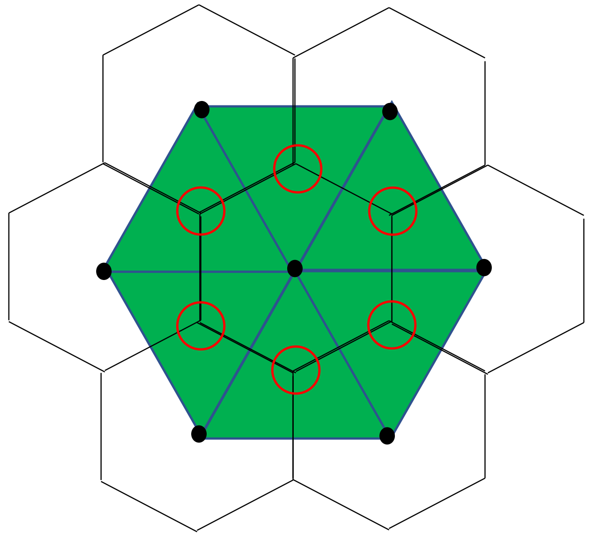

Supplementary Figure 7 Hexagonal lattice geometry. Black points represent source centers. A hexagonal unit (green) can be defined as a triangle formed by three adjacent sources. Red circles are minimum dose areas when tumor tissues are homogeneous. The spacing between sources is ~4 mm.
